# Brain and eye involvement in McCune-Albright Syndrome: clinical and translational insights

**DOI:** 10.3389/fendo.2023.1092252

**Published:** 2023-05-19

**Authors:** Ilaria Mascioli, Giulia Iapadre, Diletta Ingrosso, Giulio Di Donato, Cosimo Giannini, Vincenzo Salpietro, Francesco Chiarelli, Giovanni Farello

**Affiliations:** ^1^ Department of Pediatrics, University of Chieti, Chieti, Italy; ^2^ Department of Pediatrics, University of L’Aquila, L’Aquila, Italy

**Keywords:** McCune-Albright Syndrome, fibrous dysplasia of bone, brain, eye, *GNAS*

## Abstract

McCune-Albright Syndrome (MAS) is a rare mosaic (post-zygotic) genetic disorder presenting with a broad continuum clinical spectrum. MAS arises from somatic, activating mutations in the *GNAS* gene, which induces a dysregulated Gsα-protein signaling in several tissues and an increased production of intracellular cyclic adenosine monophosphate (cAMP). Overall, MAS is a rare disorder affecting less than 1/100,000 children and, for this reason, data establishing genotype-phenotype correlations remain limited. Affected individuals clinically present with a variable combination of fibrous dysplasia of bone (FD), extra-skeletal manifestations (including *cafeí-au-lait* spots) and precocious puberty which might also be associated to broad hyperfunctioning endocrinopathies, and also gastrointestinal and cardiological involvement. Central nervous system (CNS) and eye involvement in MAS are among the less frequently described complications and remain largely uncharacterized. These rare complications mainly include neurodevelopmental abnormalities (*e.g*., delayed motor development, cognitive and language impairment), CNS anomalies (*e.g*., Chiari malformation type I) and a wide array of ophthalmological abnormalities often associated with vision loss. The pathophysiological mechanisms underlying abnormal neurological development have not been yet fully elucidated. The proposed mechanisms include a deleterious impact of chronically dysregulated Gsα-protein signaling on neurological function, or a secondary (damaging) effect of (antenatal and/or early postnatal) hypercortisolism on early pre- and post-natal CNS development. In this Review, we summarize the main neurological and ophthalmological features eventually associated with the MAS spectrum, also providing a detailed overview of the potential pathophysiological mechanisms underlying these clinical complications.

## 1 Introduction

McCune-Albright Syndrome (MAS) is a rare mosaic disorder of striking complexity presenting with a broad clinical spectrum. Estimated prevalence ranges between 1/100,000 and 1/1,000,000, although established epidemiological data are not available (1). The original description of fibrous dysplasia (FD)/MAS in 1936 included a “classic triad” of fibrous dysplasia of bone (FD), cafeí-au-lait macules and precocious puberty (PP) (2). However, it is now recognized that the phenotype is more complex (3). MAS arises from somatic, activating mutations in the *GNAS* gene, which induces a dysregulated Gsα-protein signaling in several tissues and an increased production of intracellular cyclic adenosine monophosphate (cAMP). Importantly, somatic gain-of-function mutations of *GNAS* lead to mosaic activation of stimulatory G protein (Gsα), resulting in disease that may involve any part of the skeleton, and may be variably associated with a great number of extra-skeletal features. The original extra-skeletal manifestations of FD reported by McCune and Albright were cafeí-au-lait spots, precocious puberty, and hyperthyroidism. With time, other manifestations have been added to the spectrum of clinical features associated to FD. These included others hyperfunctioning endocrinopathies, such as growth hormone (GH) excess, hypercortisolism (and hypophosphatemia/osteomalacia). Additionally, some affected individuals also have gastrointestinal involvement, cardiac involvement, and other features (4–8).

Neurological involvement is relatively rare in individuals affected with MAS, although the association between craniofacial FD and neuro-ophthalmological manifestations, such as reduced vision and type I Chiari malformation, are well defined. Moreover, a minority of patients present some neurodevelopmental impairment, but the underlying mechanisms has yet to be fully elucidated. In this work, we reviewed the main neurological and ophthalmological features that are potentially associated with the FD/MAS spectrum, also providing a clinical and translational-based overview of the possible molecular and pathophysiological mechanisms underlying these complications.

## 2 Diagnostic criteria and clinical description

The classical definition of MAS was based on the presence of FD associated with at least one extra-skeletal manifestation; however, the diagnosis is currently based on the presence of 2 or more characteristic features. The reason to not require FD for the diagnosis of MAS is the results of ongoing researches in the field showing the better understanding of the complex molecular pathogenesis of the disorder (9). To date, the clinical features required for the diagnosis of MAS are at least two among: (*i*) fibrous dysplasia of bone, (*ii*) cafeí-au-lait skin pigmentation, (*iii*) gonadotropin-independent precocious puberty (resulting from recurrent ovarian cysts in girls and autonomous testosterone production in boys), (*iv*) thyroid lesions with or without non-autoimmune hyperthyroidism, (*v*) growth hormone excess, (*vi*) neonatal hypercortisolism (3).

Isolated monostotic bone lesions in the absence of extra-skeletal features include a broad differential diagnosis and diagnostic uncertainty, thus generally requiring biopsy and molecular testing for the MAS gene (10). The chance of detecting the pathogenic mutation is correlated with the degree of mosaicism in the examined tissue or sample, and the sensitivity of the technique (11). Testing a DNA sample from the involved tissue (*e.g*., lesional tissue) has the highest clinical sensitivity and detection rates (greater than 80%). Conversely, diagnostic rate is approximately ~20–30% when examined the DNA extracted from peripheral blood lymphocytes (12). Although detection of a pathogenic GNAS mutation may be helpful in establishing the diagnosis, a negative result does not exclude the diagnosis FD/MAS in presence of suggestive clinical features.

## 3 Etiology and pathophysiology

MAS arises from pathogenic variants in GNAS, located on chromosome 20q13.3 (9). The GNAS complex locus encodes the alpha-subunit of the stimulatory G protein (Gsα), a ubiquitous signaling protein that mediates the actions of several hormones, neurotransmitters, and paracrine/autocrine factors.

**Figure 1:**
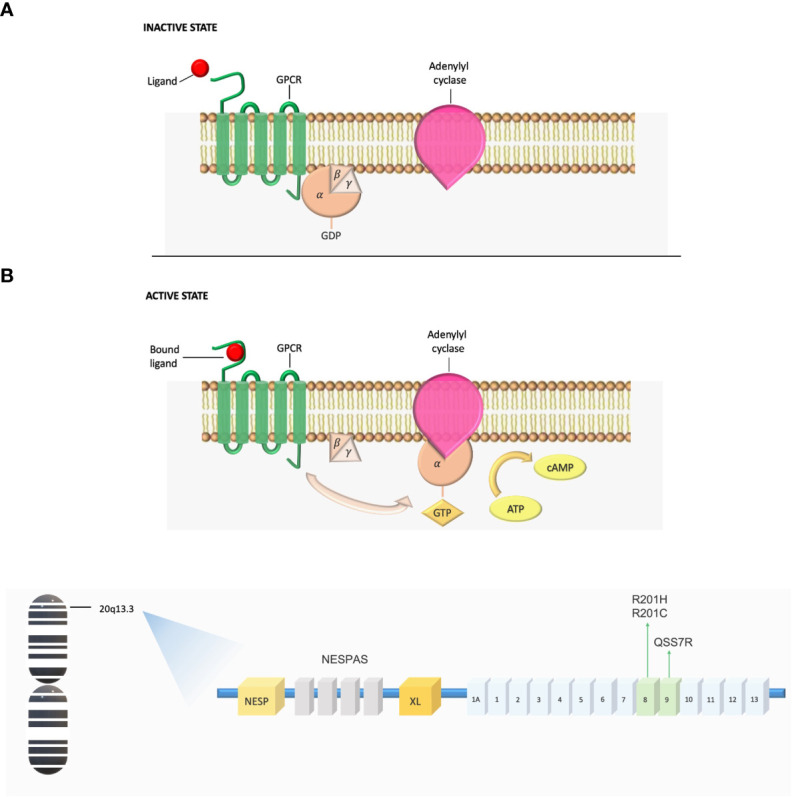
**(A)** Gs G-protein-coupled signaling dysregulation in FD/MAS. In the inactive state, the αβγ-heterotrimer is bound to GDP. After ligand binding, the GTP-bound α-subunit dissociates from the βγ-complex and activates adenylyl cyclase, leading to production of intracellular cyclic AMP and activation of protein kinase A and other downstream signaling pathways. ATP, adenosine triphosphate; cAMP, cyclic adenosine monophosphate; GDP, guanosine diphosphate; GPCR, G-coupled protein receptor; GTP, guanosine triphosphate. **(B)** Schematic of the GNAS complex locus. GNAS exons 1-13 encode Gsα. MAS arises from mutations in exon 8, where arginine 201 is converted to histidine (R201H) or cysteine (R201C), and mutations in exon 9, where glutamine is substituted for arginine (Q227R).

Gsα belongs to a class of activating Gα subunits that works by upregulating cyclic AMP (cAMP) production by adenylyl cyclase and activating protein kinase A. Gsα exists in a GDP-bound form at the basal state as part of a heterotrimeric complex. Agonist binding to Gsα-coupled transmembrane receptor promotes GDP release from the Gα subunit, allowing GTP to bind and thereby activate the G protein. Gsα is a GTP hydrolase and this activity ensures that the activation of Gsα is short-lived by converting them from the active GTP-bound conformation to the inactive GDP-bound conformation (13) (**Figure 1A**).

Over than 95% of Gsα mutations in FD/MAS occur at exon 8, in which the arginine 201 is converted to either a histidine (R201H) or a cysteine (R201C); very infrequently, arginine is replaced by serine, glycine, or leucine. Most of the remaining pathogenic mutations occur in codon Q227 within exon 9, where a Glutamine residue is replaced with Arginine (Q227R) (14, 15). Pathogenic *MAS* variants at either codon inhibit the GTPase activity of Gsα leading to constitutively activated cAMP signaling and resulting in prolonged Gsα active GTP-bound state (16) (**Figure 1B**).

GDP-bound Gsα is inactive under normal physiologic conditions, however recent evidence demonstrates that GDP-bound Gsα bearing the R201C mutation is in an active conformation under physiologic conditions and can activate adenylyl cyclase (17). Germline *gain-of-function* mutations have never been reported and putatively considered to be incompatible with life. Thus, the pathogenic variants underlying the FD/MAS spectrum occur post-zygotically at an early embryogenesis stage. During development, mutant progeny cells migrate to different parts of the skeleton and extra-skeletal tissues, resulting in mosaic expression of constitutively active Gsα. The resulting phenotype is largely determined by the timing of the mutational event during embryogenesis and the extent and location of mutation-bearing tissue (18).

## 4 Neuro-ophthalmological features

### 4.1 Chiari I malformation and basilar invagination

Importantly, FD lesions can involve any part of the skeleton. However, the skull base is among the most affected regions. Craniofacial FD typically presents with painless “bump” and facial asymmetry, and asymptomatic lesions are often detected incidentally on brain imaging studies (19). However, neurological complications from skull base deformities have been reported (20, 21).

Chiari I malformation (CM1), characterized by an extension of the cerebellum below the foramen magnum, and secondary basilar invagination (BI), in which the odontoid prolapses into the posterior cranial fossa, represent two potentially serious complications of metabolic bone disorders (22, 23). CM1 and BI occurred in 6.3% and 7.6% of patients with craniofacial FD, respectively (24).

Basilar invagination occurs due to distortion of the craniovertebral junction, secondary to bone weakness, resulting in rostral malposition of the cervical spine (25).

While the causal mechanism of BI in metabolic bone disorders is well described, multiple causal mechanisms have been implicated CM1 and, as a result, the CM1-related pathophysiological basis is highly heterogeneous. However, in both CM1 and BI, there is a functional narrowing of the foramen magnum which may result in life threatening compression of the cerebellum and spinal cord.

Notably, FD lesions are characterized by a tendency to bone expansion. In this context, cranial constriction and cranial settling are considered to be the most prominent mechanisms for CM1 development in individuals affected with FD/MAS (26).

Additionally, increased intracranial pressure is another proposed mechanism underlying CM1 (27). The resulting CM1-related intracranial hypertension might be in turn exacerbated by impairment of venous drainage at the foramen magnum, leading to accumulation of cerebrospinal fluid (CSF) manifesting as hydrocephalus.

CM1 commonly occurs in association with BI (23), as the malposition of the odontoid process can result in a refractory downward shift of the cerebellar tonsils (28).

The contribution of MAS-related endocrinopathies in the development of CM1 and BI has not been fully elucidated. However, FGF23-mediated hypophosphataemia and hyperthyroidism may contribute to bone fragility and increase the risk of developing complications such as MC1 and BI.

The clinical presentations of CM1 and BI are similar. The most common symptoms, such as headache, pain, paresthesia and visual deficits are frequently observed in craniofacial FD. In contrast, symptoms more specific for brainstem compression are uncommon in craniofacial FD (10). Patients may present with dissociative sensory loss, loss of pain and temperature sensation, and absence of gag reflex (29).

When these atypical sings are found in patients with FD/MAS, clinicians should suspect and investigate the presence of complications such as CM1 and BI.

### 4.2 Optic disc edema

Optic disc edema (ODE) is a serious ophthalmologic condition which has been occasionally reported in individuals affected with MAS (30, 31). However, the etiology of MAS-related ODE has not been clearly defined. Potential mechanisms include intracranial compression, CSF flow alterations (due to skull base deformities), space-occupying lesions, as well as an increased intracranial pressure secondary to other associated hyperfunctioning endocrinopathies (32, 33).

In a recent study evaluating individuals affected with MAS as part of the National Institutes of Health (NIH) cohort, craniofacial FD was found to be a risk factor for ODE, with a prevalence of 3.7%. In most cases, ODE was observed in children and adolescents aged less than 18. Furthermore, all cases were reported to be mild and non-progressive (34). Craniofacial deformities are common in FD/MAS and skull base involvement may result in increased intracranial pressure. As mentioned above, CM1 may lead to intracranial hypertension due to obstruction of cerebrospinal fluid flow at the foramen magnum (35) and, according to some studies, there was a strong association between ODE and CM1 (34).

Rarely, individuals affected FD/MAS may also develop secondary aneurysmal bone cysts, with a reported prevalence of approximately 5% (36). Aneurysmal bone cysts are expansive, fluid-filled lesions that act as space-occupying lesions resulting in increased intracranial pressure and ODE.

Furthermore, the NIH cohort evaluation revealed a positive correlation between leuprolide therapy and ODE. Leuprolide is a synthetic analog of gonadotropin-releasing hormone (GnRH) used for the treatment of central precocious puberty in children. Although peripheral precocious puberty is among the most common forms of precocious puberty in MAS, progression to central puberty requiring GnRH analogue treatment is possible (37). In the past, several studies reported the association between leuprolide and intracranial hypertension both in children and adults (38, 39). However, the mechanism underlying the increased intracranial pressure has not been fully elucidated.

### 4.3 Vision loss in MAS

Vision loss is an uncommon but serious complication of craniofacial FD. Tendency to bone expansion may result in deformation of the optic canals and compression of nerve fibers resulting in variable degrees of vision loss (40, 41).

Symptomatic patients with optic nerve involvement often undergo decompression surgery; however, there is variable efficacy in attempt to restore visual function or to alleviate symptoms (42, 43).

Visual impairment in patients with FD/MAS cannot be explained purely by physical nerve compression itself (44, 45). Additional factors may also facilitate visual impairment, and these include structural and/or functional anomalies within central visual pathways. Several individual case studies noted a loss of with matter fiber density within the optic radiations and functional changes involving the primary visual cortex (46). It has been recently suggested that the use of advanced imaging techniques characterizing CNS white matter pathways and visual cortex function would better explain the role of central (*i.e.*, primary visual cortex) anomalies in visual loss of individuals affected with FD/MAS (46).

### 4.4 Neurodevelopmental disorders as part of the FD/MAS spectrum

Intellectual disability of variable degrees of severity has been described many decades ago in association with FD/MAS (47), and over the years several studies have confirmed such association.

Some interesting data emerged from the evaluation of the NIH cohort by Brown et al. (48). Interestingly, up to 9% of the entire patient cohort was found to have some neurodevelopmental impairment including cognitive and learning difficulties, speech delay or apraxia and/or delay of motor developmental milestones. Importantly, a large proportion of children affected with Cushing’s syndrome usually have some neurodevelopmental impairment with both cognitive and motor delay, implicating MAS-related glucocorticoid excess as a potentially important risk factor for neurodevelopmental features in individuals affected with FD/MAS. In fact, an increased risk for neurodevelopmental impairment (> 40%) has been reported in individuals affected presenting glucocorticoid excess in the context of a diagnosis of FD/MAS (48).

#### 4.4.1 Glucocorticoids excess in FD/MAS and impaired neurological function

Optimal levels of glucocorticoids are required for neuronal growth, differentiation and survival, as well as an adequate synaptic plasticity. Neurological function is impaired by high levels of glucocorticoids due to their crucial role in brain development and their influence on cognition, behavior and early life programming of stress reactivity. As shown in both animal and human studies, prenatal exposure to glucocorticoid excess, may lead to permanent behavioral changes (49–51). Glucocorticoids influence the development of brain regions crucial to learning and memory processes, such as cortex, and amygdala (52). Prolonged hypercortisolemia is associated with impairment of specific domains of cognition, in particularly verbal function such as verbal learning (53, 54). Hypercortisolemia may induce alterations in the development of specific additional brain target areas, as demonstrated by functional imaging studies showing an association between chronic hypercortisolemia and reduced hippocampal volume (55).

Neonatal-onset hypercortisolism is among the rarest MAS-related endocrine features, occurring in up to 7% of affected individuals, and it has been associated with poorer clinical outcomes including an increased risk of early mortality (48). Hypercortisolism typically presents during the first year of life with a median age at diagnosis of 3 months (48–56).

Since hypercortisolism occurs early, it has been hypothesized that developmental problems among Cushing’s syndrome survivors are due to excess of glucocorticoids during pre- or early post- natal stages of life (48).

Notably, studies in adult individuals affected with Cushing’s syndrome report a high incidence of neuropsychiatric diseases, most commonly depression and/or major affective disorders, and these complications are usually reversible after treatment (57). Studies among pediatric patients suggest that exposure to endogenous glucocorticoids excess is associated with a decline in cognitive performance (58).

However, the effects of long-term exposure to elevated endogenous glucocorticoids on neurological function and the pediatric developing brain, have not been fully elucidated in clinical or basic science studies. In the study by Keil et al. association between the age at onset of hypercortisolism and the effects on cognitive outcomes after treatment was evaluated; importantly, children that developed Cushing syndrome at later stages of life experienced less decline in cognitive function than children who had a neonatal or early infantile- onset of the condition (59).

Experimental data from animal studies exposed to perinatal glucocorticoids confirmed adverse effects on cognitive function (60, 61). In contrast, cognitive function in infants at risk of congenital adrenal hyperplasia or preterm birth who were exposed to glucocorticoids *in utero* did not show significant differences in cognitive outcomes related to glucocorticoid exposure (62, 63).

Thus, the difference may be due to the higher doses used in animal experiments than those used for treatment purposes. Similarly, children with MAS and associated hypercortisolism may have been exposed due to their condition to levels of glucocorticoids higher than those used therapeutically (for pulmonary maturation and congenital adrenal hyperplasia).

#### 4.4.2 GNAS pathogenic variants and the developing CNS: insights from translational studies

An alternative explanation for the presence of neurological and neurodevelopmental features in individuals affected with FD/MAS is the chance of activating mutations of the Gsα protein within the CNS as part of the mosaic distribution; survivors of Cushing’s syndrome may be more affected due to greater total body mutation burden.

The cAMP cascade modulates an array of processes within the central nervous system, including learning and memory. Acute activation of Gαs stimulates the production of adenyl cyclase (AC), thereby increasing cyclic AMP (cAMP) levels. Chronic stimulation of Gαs, however, triggers a compensatory degradation of protein kinase A (PKA) dependent cAMP by phosphodiesterases (PDEs) in selected brain regions (64).

PKA, a serine/threonine kinase, is the major receptor for cAMP in mammals, formed by a dimer of two regulatory (R) subunits that each binds a catalytic subunit (C). R subunits contain two cAMP binding sites; cAMP occupation leads to conformational changes of the R subunits and release of the active C subunits (65).

**Figure 2:**
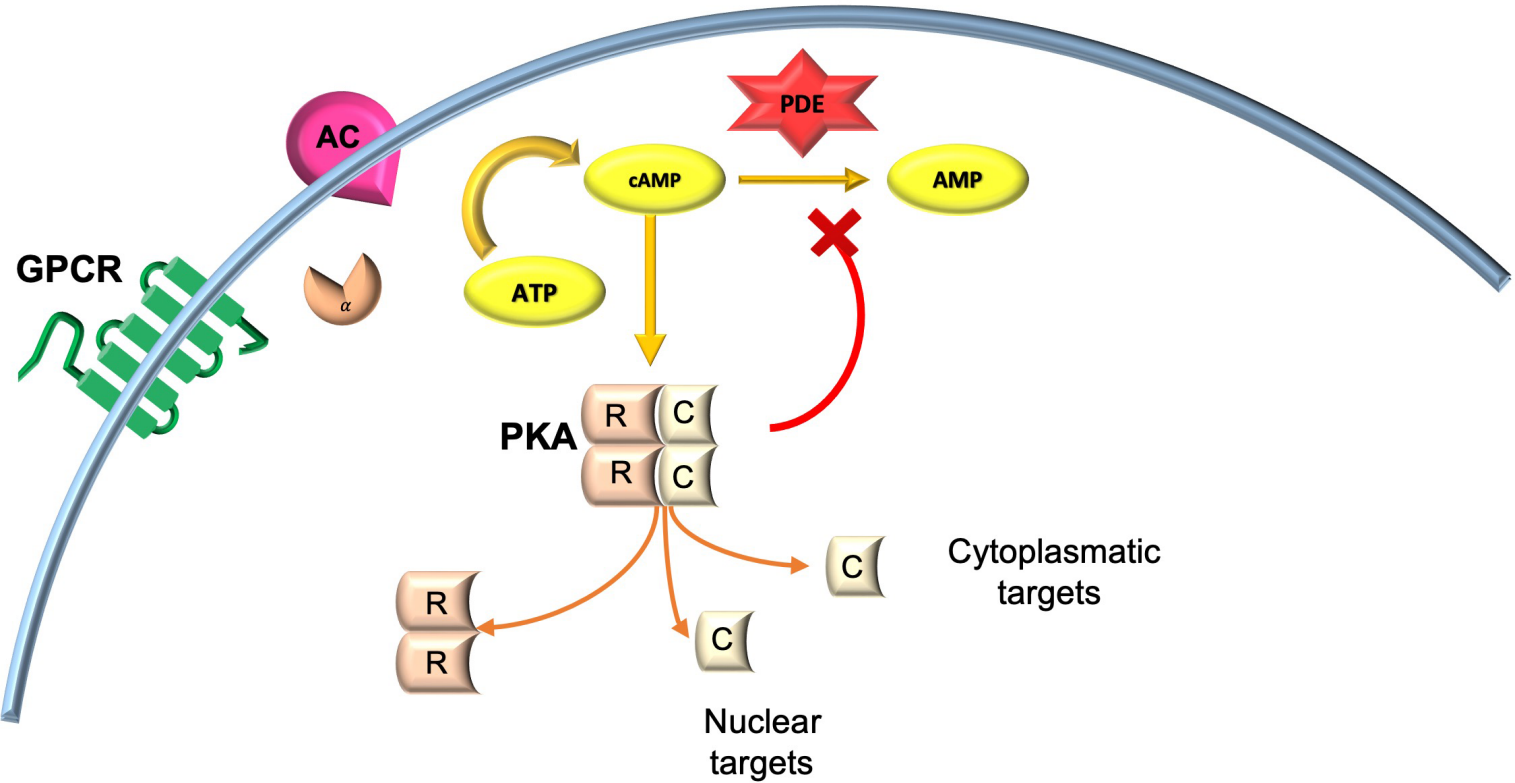
cAMP/PKA signaling in neuronal cells. AC, adenylyl cyclase; AMP, adenosine monophosphate; ATP, adenosine triphosphate; cAMP, cyclic adenosine monophosphate; GPCR, G-coupled protein receptor; PKA, protein kinase A.

cAMP signaling, following chronic activation of Gαs, is turned off by phosphodiesterase (PDE)-catalyzed conversion of cAMP to AMP (66). Chronic activation of PKA in turn upregulates PDE activity, with consequent degradation of the cAMP and cessation of the signal (**Figure 2**).

Genetic, pharmacological and electrophysiological studies on different animal models have highlighted the importance of the cAMP/PKA pathway in synaptic plasticity, learning and memory (67–70).

As extensively described by Lee D, cAMP acts on CNS neurons by modulating cellular excitability. In particular, a dual regulatory role of cAMP has emerged. It increases overall presynaptic function globally on the one hand, but it acts locally on postsynaptic GABA receptors to decrease GABAergic plasticity. Thus, the action of cAMP results in further increases in neural excitability (71).

Studies in transgenic mice in which the activating mutation p.Q227L of Gsα, functionally similar to the R201C/H mutations that cause FD/MAS, was targeted at the central nervous system (Gsα * Q227L) showed that Gsα* mice exhibit a neurobehavioral phenotypes including short- and long-term memory deficits and impaired spatial and associative learning (72, 73). Gsα* mice show significant increases in adenylyl cyclase activity in cortex, hippocampus and striatum (74). As predicted by this increased cyclase activity, Gsα* transgenic mice show significantly increased cAMP levels in the striatum; however, in the cortex and hippocampus, Gsα* mice show significantly reduced cAMP levels due to PKA-dependent compensatory upregulation in total cAMP PDE activity (75).

Similarly, previous studies on *Drosophila Melanogaster* documented that both increased and reduced levels of adenylate cyclase could impair learning and/or olfactory memory (76). Behavioral deficits caused by Gsα overexpression appear to be due to compensatory decreases in cAMP levels and are reversible by pharmacologically increasing cAMP signaling. Studies in mice show that injections of PDE inhibitors, which increase cAMP signaling, facilitate the establishment of long- term memory (70–78). It is worth noting whether phosphodiesterase inhibitors may be helpful in those patients with significant cognitive or neurodevelopmental dysfunction. However, phosphodiesterase inhibitors could theoretically worsen other manifestations of MAS, whose pathophysiology is due to the excess of cAMP (79).

## Conclusion

FD/MAS is a rare disorder of striking clinical and translational complexity. Neurological and ophthalmological involvement is a rare but serious complication of the condition. Although, craniofacial FD is an important risk factor for the onset of neurological complications, the role of MAS-related hyperfunctioning endocrinopathies and associated therapies as neurological risk factors has yet to be fuly understood. Isolated CNS involvement is possible, however the role of activating mutations of the Gαs protein within the developing/pediatric brain remains to be clarified. Further clinical and basic science-based studies will be needed to full address these research questions and to understand the pathophysiological impact of FD/MAS on brain and eye development and function.

## Author contributions

IM wrote the manuscript. VS, CG and GD reviewed the paper. All authors contributed to the articleand read and approved the submitted version.
